# South-West Orthopaedic Club

**Published:** 1971-04

**Authors:** 


### South-West Orthopaedic Club

A meeting of the Club was held at the Prince of Wales Orthopaedic Hospital, Cardiff, on the 28th November, 1970.

**Orthopaedic Aspects of Behcet's Syndrome** — Mr. H. J. Hambury (Swansea) demonstrated the case of a man of thirty-eight, presenting with a bilateral knee synovitis associated with buccal and genital ulceration, who was diagnosed as having Behcet's syndrome. This syndrome is thought to be connected with a disturbance of fibrinolytic activity, and in this particular case there was an increase in the euglobulin lysis time and an increase in the fibrinogen content of the serum. Phenformin and ethyloestranol increase fibrinolytic activity and their exhibition led within a week to healing of the buccal and scrotal ulceration and complete regression of the synovitis.

**Traumatic Pneumonopathy** (guest lecture) — Mr. Hugh Harley (Cardiff) gave an excellent account of the abnormal respiratory features which may be associated with thoracic injuries.

**Leg Equalization by Epiphyseal Arrest 1952-1970** — Mr. J. G. H. James (Cardiff and Neath) reviewed a personal series of 246 cases of leg disparity: 114 had been followed to maturity and 67 cases of epiphyseal stapling were analysed in detail. The intention was to staple one or both epiphyses under direct vision with accurate placement of the posterior femoral and lateral tibial staples particularly, disregarding the fibula and leaving the staples in situ. The residual disparity was less than 1 cm. in 69%, between 1 and 2 cm. in 17%, totalling 86% with under 2 cm. disparity. A second operation was necessary in 8 cases (12%) — 5 for extrusion or breaking of steel staples and 3 for unacceptable varus deformity requiring corrective osteotomy. Of previously recorded complications, subjective symptoms and scarring were insignificant, as was cruciate laxity, but a small proportion developed collateral laxity or genu recurvatum. Improvement in these results should be expected with use of vitallium staples since 1962 and more accurate prediction in different ethnic groups.

**Disturbed Epiphyseal Growth in the Region of the Knee after Osteomyelitis in Infancy** — Mr. P. H. Roberts (Weston-super-Mare) gave an account of fifteen patients who had had osteomyelitis of the lower femur or upper tibia during infancy. Deformity developed to some extent in all cases and appeared early, and in most patients required operative treatment for its correction. Shortening of the limb occurred in all patients and in some was severe. Epiphyseal damage might be due to an abscess or to ischaemia. In the early stages the radiographic appearances could be deceptive, suggesting that damage to the epiphysis was irreparable. However, significant recovery of the epiphysis could occur after a delay of several years and this, together with the often good function which is preserved at the joint, should deter the surgeon from early destructive operation on an affected limb.

**Reconstruction of the Leprosy Hand** (film) — Mr. W. M. Lennox (Gloucester).

**Lesions in the Capitellum** — Mr. H. Weisl (Cardiff) gave an account of eleven early cases of osteochondritis dissecans of the elbow in adolescents. Each patient presented radiologically with a subchondral radiolucent area in the capitellum. In eight of these cases it was possible to observe the formation of a loose body. In one case, healing of the radiolucent area without loose body formation was observed. This suggests that the radiolucent lesion in the capitellum precedes the formation of loose bodies and that this lesion occasionally heals without loose body formation. In most cases the loose bodies which had formed remained symptomless. Three of the eleven elbows were explored. The patients whose elbows had been explored had a greater limitation of elbow extension than those who were treated solely by conservative measures. This confirms the value of the established policy of conservative management of osteochondritis dissecans of the elbow.

**Osteomyelitis of the Spine** — Mr. J. Hombal (Cardiff reviewed 67 cases of osteomyelitis of the spine, drawing particular attention to the diagnostic features. Thirty-five were initially mistaken for tuberculosis and the sub-acute variety can be difficult to diagnose. Over a quarter had abnormal neurological signs, but only two patients had spinal cord involvement. "Parrot beaking" due to unequal bone formation under the longitudinal ligaments is a diagnostic radiological sign, but was present in less than half the cases. A more reliable sign is the rapid destruction of the disc space, often taking place within four weeks. Over three quarters of the cases did not achieve bony fusion.

**Fat Embolism** — Mr. J. D. M. Blayney (Cardiff) gave a review of the up-to-date knowledge available of this condition.

**Erythroedema** — Mr. H. Harp-Griffiths (Newport) drew attention to a type of neuropathy which he had recently been able to distinguish in which erythroedema of the limbs occurred in association with painful peripheral neuropathy. Associated clinical features were watery eyes, a pink facies and pink palms. He believed that an awareness of this clinical picture would lead to its wider recognition. The aetiology, however, was unknown.

**A Brief Visit to Russia** — Mr. H. J. Rogers (Cardiff) gave an account of a recent visit he made to Russia.

